# An Online Hashing Algorithm for Image Retrieval Based on Optical-Sensor Network

**DOI:** 10.3390/s23052576

**Published:** 2023-02-25

**Authors:** Xiao Chen, Yanlong Li, Chen Chen

**Affiliations:** 1 Department of Information and Communication, Guilin University of Electronic Technology, Guilin 541004, China; 2 Ministry of Education Key Laboratory of Cognitive Radio and Information Processing, Guilin University of Electronic Technology, Guilin 541004, China

**Keywords:** optical-sensor network, manifold learning, balanced similarity, discrete binary optimization, image retrieval

## Abstract

Online hashing is a valid storage and online retrieval scheme, which is meeting the rapid increase in data in the optical-sensor network and the real-time processing needs of users in the era of big data. Existing online-hashing algorithms rely on data tags excessively to construct the hash function, and ignore the mining of the structural features of the data itself, resulting in a serious loss of the image-streaming features and the reduction in retrieval accuracy. In this paper, an online hashing model that fuses global and local dual semantics is proposed. First, to preserve the local features of the streaming data, an anchor hash model, which is based on the idea of manifold learning, is constructed. Second, a global similarity matrix, which is used to constrain hash codes is built by the balanced similarity between the newly arrived data and previous data, which makes hash codes retain global data features as much as possible. Then, under a unified framework, an online hash model that integrates global and local dual semantics is learned, and an effective discrete binary-optimization solution is proposed. A large number of experiments on three datasets, including CIFAR10, MNIST and Places205, show that our proposed algorithm improves the efficiency of image retrieval effectively, compared with several existing advanced online-hashing algorithms.

## 1. Introduction

With the popularization of optical-sensor networks and the wide use of intelligent interconnected devices, data in various fields are increasing at an unbelievable speed. People realize that intelligent processing and analysis of data is necessary [1,2,3] and it is of great significance to store high-dimensional data effectively and retrieve data rapidly. The traditional indexing methods involving text-based image retrieval (TBIR) and content-based image retrieval (CBIR) [4,5] encounter the curse of dimensionality in high-dimensional situations, and their query performance is even worse than linear query. An approximate nearest-neighbor query based on the hash method is an efficient method to solve the above issue [6,7]. Specifically, in image retrieval systems, image hashing means mapping a high-dimensional real-valued image to a compact binary code, which can preserve the relationship between different high-dimensional data and the Hamming space [8,9,10,11].

According to the dependency between the hash model and the sample data, hashing algorithms include data-independent algorithms and data-dependent algorithms. The representative data-independent algorithms include locality-sensitive hashing (LSH) [12,13], and its variants such as $\ell_{p}—$stable hashing [14], min-hash [15], and kernel LSH (KLSH) [16]. Data-dependent hashing algorithms include unsupervised hashing algorithms [17,18,19] and supervised hashing algorithms [20,21,22,23]. Since methods using data distribution or class labels perform better in the quick search field, more effort is being put into data-dependent methods.

In fact, data samples always arrive sequentially, as time goes on, and thus the previously existing data is often out of date [24,25]. When the discrepancy between newly arrived data and previously existing data is large, the hashing function often loses efficiency on newly arrived data. Therefore, it is very important to present an online hashing model that is suitable for streaming data. Unlike the offline hashing methods, which correct the training error on the fixed dataset through multiple training rounds, online algorithms use multiple batches of streaming data to update hash functions, which are more realistic and have a strong application background [26,27,28,29,30,31].

Several classic online hashing methods have emerged in recent years, and they are all data-dependent. Representative works include Online Hashing (OKH) [24], Adaptive Hashing (AdaptHash) [32], Online Supervised Hashing (OSH) [33], Online Hashing with Mutual Information (MIHash) [34], Balanced Similarity for Online Discrete Hashing (BSODH) [35], Supervised Online Hashing via Hadamard Codebook Learning (HCOH) [36], Hadamard Matrix Guided Online Hashing (HMOH) [37], etc.

Most of the mentioned online-hashing algorithms consider the adaptability, pairwise similarity, or independence of hash codes to build a constrained hashing model, but the optimization needs relaxation learning, which brings quantization errors to a certain extent and reduces the retrieval accuracy. In addition, there exist unsupervised online hashing methods, which are mostly based on the idea of “matrix sketch”, and its representative works mainly consist of Online Sketching Hashing (SketchHash) [38], Faster Online Sketching Hashing (FROSH) [39], and so on. From the setting of online hashing, the global data grow dynamically with the current arriving data, which just represent the local data at a certain stage. Unsupervised methods that only rely on the distribution of newly arrived data lack a global description of the hashing model.

In image-retrieval application systems, the labeling is carried out manually and the workload is also huge. In addition, manual labeling is prone to errors, and wrong labels will directly lead to retrieval failure. Therefore, the online hashing method that relies too much on data labels while ignoring the structural characteristics of the data itself is subject to many limitations in practical applications, which seriously affects the performance of retrieval accuracy [40,41].

The high-dimensional image data remains on the low-dimensional manifold structure [42], and the query data is often strongly correlated. Therefore, this paper proposes an online hashing model that fuses global and local dual semantics. First, an anchor hash model is built based on manifold learning to retain local features of the original data. Then, a global similarity matrix which is used to constrain the hash codes is constructed, employing the balanced similarity between newly arrived data and previous data [35]. Under a unified framework, an online hash model integrating global and local dual semantics is learned, as well as an effective discrete-binary-optimization scheme being proposed. Compared with several classical and well-established online hashing algorithms, our proposed LSOH method has advantages in many performance indicators.

In summary, the main contributions of our work are as follows:

- Extract the manifold structure of high-dimensional data using Laplacian Eigenmaps, thus constructing an anchor hash model.
- Construct an asymmetric-graph regularization term to constrain the learning process of hash codes using the balanced similarity between current arriving data and previous data sets.
- Integrate the anchor hash model and the asymmetric graph regularization with a seamless formulation to learn global and local dual-semantic information, then use the alternating-iteration algorithm to solve the optimization issue and obtain high retrieval accuracy by performing a large number of experiments.

The remaining contents are arranged as follows. In Section 2, related work in this field is reviewed. In Section 3, we present our proposed online-hashing algorithm, including the optimization method. Section 4 presents the experimental results and analyses in detail. Finally, we give a conclusion of our work in Section 5.

## 2. Related Work

In this section, online hashing algorithms, such as OKH [24], AdaptHash [32], OSH [33], MIHash [34], BSODH [35], HCOH [36], HMOH [37], SketchHash [38] and FROSH [39] are introduced. Among them, all are supervised hashing methods except SketchHash and FROSH. Supervised hashing is more efficient owing to the semantic data, but online retrieval datasets are often prone to missing labels and labeling errors, while hashing methods without labels are more suitable for massive online-retrieval applications.

Huang et al. [24] presented an online hashing algorithm using a kernel function, termed OKH. First, OKH employes the kernel-based hash function to process linearly inseparable data. Then, OKH formulates an objective function based on the inner product of binary codes. They consider the equivalence between optimizing the inner product of binary codes and Hamming distance, and use the greedy algorithm to solve the hash function effectively. It solves the non-convex optimization problem of Hamming distance. Experiments show that it can be widely applied to image-retrieval scenarios.

Similar to OKH’s framework, AdaptHash [32] proposes a fast similarity-search algorithm for hash functions, based on the stochastic gradient descent method. Specifically, it defines a hinge-loss function to determine the number of hash functions that need to be updated in Adaptive Hash and optimizes the model by SGD.

Cakir et al. [33] proposed an adaptive online-hashing method based on Error Correcting Output Codes (ECOC), named OSH. No prior assumptions about label space are made and it is the first supervised hashing algorithm suitable for the growth of label space. OSH presents a two-step hashing framework, first generating ECOC as codebooks, and then assigning codewords to each class label. Finally, the exponential loss is optimized and solved by SGD, to ensure that the learned hash function is suitable for binary ECOC.

Based on the knowledge of information theory, MIHash [34] takes mutual information as the learning objective and proposes a measure to eliminate the updates of the unnecessary hash tables. Thus, they optimize the mutual information objective by stochastic gradient descent. The computational complexity is effectively reduced, and the learning efficiency of the hash function is improved.

BSODH [35] believes that there are two unsolved problems: update imbalance and optimization inefficiency, which lead to the unsatisfactory performance of OH in practical applications. In this paper, two balance parameters are introduced to improve the regularization term of asymmetric graphs. Theoretical analysis and extensive experiments verify the role of parameters in alleviating the unbalanced update. It is also the first time discrete optimization has been applied to online hashing, which improves the online hashing performance.

Lin et al. [36] believe that because of the flaw of unknown category numbers in supervised learning, it does not improve the efficiency of online hash retrieval, despite the addition of semantic information. Therefore, they propose a robust supervised online- hashing scheme, termed HCOH. First, a high-dimensional orthogonal binary matrix, i.e. the Hadamard matrix, is generated. Every column or row of this matrix can be taken as a codebook that corresponds with a class label. Then, LSH is used to convert the codebook into a binary code adapted to the number of hash bits. In an improved version of HMOH [37], hash linear regression is processed as a binary-classification issue, and the case of multi-label is considered as well.

Aimed at the problem of the data embedding into the system in a streaming way and the difficulty of loading into memory for training because of the huge dataset, SketchHash [38] decreases the size of the dataset based on the idea of data sketches, and retains the main features of the dataset to learn an effective hash function. This approach reduces computational complexity and space complexity. Compared with SketchHash, the FROSH [39] method leverages fast transformation to sketch data more compactly. FROSH applies a specific transform on different small data blocks, speeding up the procedure of sketching with the same space cost.

There is also semi-supervised online hashing [43,44], which is relatively complicated because labels may come from existing data or streaming data. In addition, deep-hashing methods [40,45,46] occupy a very important position in the existing offline-hashing methods. However, there are large amounts of parameters to be trained in deep learning, and few examples are applied in online hashing at present. Among them, Online Self-Organizing Hashing [47] obtains hash codes by the Self-Organizing Map (SOM) algorithm, but SOMs with multi-layers structures have not been applied to image retrieval.

## 3. The Proposed Method

**Figure 1:**
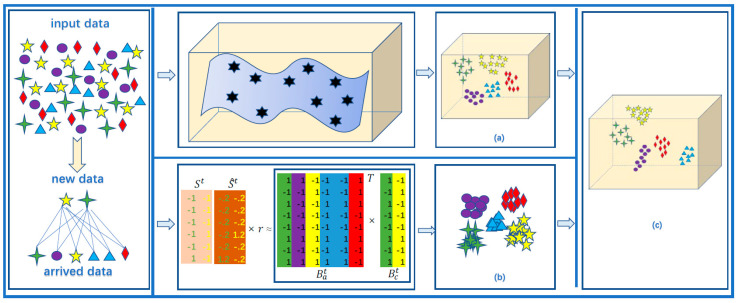
The overall framework of online hashing preserving local features and global-balanced similarity (LSOH). (**a**) Manifold learning preserves the structural features of newly arrived data, and obtains the hash codes of newly arrived data through Laplacian Eigenmaps (LE). (**b**) Learn binary codes by a balanced similarity matrix built from newly arrived data and existing data to keep all the hash codes consistent. (**c**) Our proposed algorithm can learn hash codes preserving dual-semantic information, and obtain satisfactory retrieval results.

In this section, the variable symbols in this algorithm are first defined, and the modeling process that combines the local structural features and the similarity features of the global datasets is given. Finally, we obtain the objective function and solve it by the alternating-iteration method. The algorithm frame is shown in Figure 1.

### 3.1. Notations

Assume that $n_{t}$ training samples are poured into retrieval application at t stage. They are denoted as $X^{t}=\left[x_{1}^{t},x_{2}^{t},\cdots ,x_{n_{t}}^{t}\right]\in R^{d\times n_{t}}$ and their corresponding labels $L^{t}$ are defined as $L^{t}=\left[l_{1}^{t};l_{2}^{t};\cdots ;l_{n_{t}}^{t}\right]\in N^{d\times n_{t}}$. Each training sample expressed as $x_{i}^{t}$ is d-dimensional. The goal of hashing is to learn r-dimensional hash codes, which are denoted as $B^{t}=\left[b_{1}^{t},b_{2}^{t},\cdots ,b_{n_{t}}^{t}\right]\in {\left\{1,-1\right\}}^{r\times n_{t}}$, and meanwhile, r is much smaller than d. The linear-hash mapping is widely used as a hash function [48], i.e.,

(1) $$B^{t}=F\left(X^{t}\right)=sgn\left({W^{t}}^{T}X^{t}\right)$$

where F(·) stands for the hash function, $W^{t}\in R^{d\times r}\;$is the projection matrix to be learned, ${W^{t}}^{T}$ is the transpose of $W^{t}$, and sgn(·) is the symbolic function, and its definition is the following. All symbol notations utilized in this study are presented in Table 1.

(2) $$f\left(x\right)=sgn\left(x\right)=\{\begin{array}{cc} -1, & x<0\\ 1, & x\geq 0 \end{array}$$

### 3.2. Manifold Learning

#### 3.2.1. Laplacian Eigenmaps

Laplacian Eigenmaps use the Laplacian operator to make similar data in the original space as close as possible after being mapped to the low-dimensional space, so as to embed high-dimensional images in the low-dimensional space. Assume that the original data denoted as $X^{t}=\left[x_{1}^{t},x_{2}^{t},\cdots ,x_{n_{t}}^{t}\right]\;$are mapped to the low-dimensional space, in which hash codes are expressed as $B^{t}$=$\left[b_{1}^{t};b_{2}^{t};\cdots ;b_{n_{t}}^{t}\right]$. We construct a graph whose adjacency matrix is $O^{t}$ to maintain the relationships between different data. Then, we define the objective function to be optimized as follows:

(3) $$\underset{B^{t}}{\min}\underset{ij}{\sum }{\left\|b_{i}-b_{j}\right\|}_{2}^{2}{O_{ij}}^{t}$$

$$s.t.\;B^{t}\in {\left\{-1,1\right\}}^{k\times n_{t}},\;\;\;{B^{t}}^{T}B^{t}=n_{t}I,\;\;\;{O_{ij}}^{t}=\exp\left(-\frac{{\left\|x_{i}-x_{j}\right\|}^{2}}{\varphi^{2}}\right).$$

where I represents the k-dimensional identity matrix, and ${O_{ij}}^{t}$ represents the adjacency matrix between the sample data. Mathematically, the objective function to be optimized can be transformed into the formula as follows:

(4) $$\underset{B^{t}}{\min}tr\left(B^{t}\left(D-O\right){B^{t}}^{T}\right)$$

$$s.t.\;B^{t}\in {\left\{-1,1\right\}}^{k\times n_{t}},\;\;\;{B^{t}}^{T}B^{t}=n_{t}I.$$

where $D_{ii}=\sum_{i}O_{ij}$ represents the weight matrix of the sample graph, and D−O is the Laplace matrix. By eigen decomposition of D−O, the eigenvectors corresponding to the k smallest non-zero eigenvalues are obtained as the required target hash codes.

#### 3.2.2. Anchor Graph Hashing

It is time-consuming and memory-intensive to compute the adjacency matrix for large amounts of sample data. Calculating the adjacency matrix by using an anchor set instead of the dataset can solve the above problem: m anchor points denoted as $\left[u_{1},u_{2},\ldots ,u_{i},\ldots ,u_{m}\right]\in R^{d}$ are obtained through the k-mean clustering method. When the number of anchors is less than that of the training samples, both storage cost and computation time are greatly reduced. The anchor graph is denoted as $A^{t}$ and its elements are defined as follows:

(5) $${A_{ij}}^{t}=\{\begin{array}{cc} \frac{exp\left(-{\left\|x_{i}^{t}-u_{j}^{t}\right\|}^{2}/\theta \right)}{\sum_{j^{\prime }\in \left\{i\right\}}exp\left(-{\left\|x_{i}^{t}-u_{jj^{\prime }}^{t}\right\|}^{2}/\theta \right)}, & \forall j\in \left\{i\right\}\\ 0, & otherwise \end{array}$$

where θ is a defined parameter, and {i} represents the index set of the k nearest anchor points. Replace the traditional Laplace matrix with anchor graph $A^{t}$, and then the objective function is obtained.

(6) $$L_{1}=\underset{{\tilde{B}}^{t},W^{t}}{\min}\overset{m}{\underset{j=1}{\sum }}\overset{n}{\underset{i=1}{\sum }}{\left\|{\tilde{b}}_{j}^{t}-{W^{t}}^{T}x_{i}^{t}\right\|}_{2}^{2}A_{ij\;,}^{t}$$

where ${\tilde{B}}^{t}=\left[{\tilde{b}}_{1}^{t},{\tilde{b}}_{2}^{t}\ldots {\tilde{b}}_{j}^{t},\ldots {\tilde{b}}_{m}^{t}\right]$ represents the hash codes of anchor points, ${W^{t}}^{T}x_{i}^{t}$ represents the hash codes of the input images, and ${A_{ij}}^{t}$ represents the anchor graph matrix constructed by the input images and anchor data.

### 3.3. Global-Balanced Similarity

It performs the anchor hashing based on Laplacian Eigenmaps; the hash function of newly arrived data is obtained independently, which will ignore the correlation of the overall data and boost the redundancy of hash codes. Thus, a global similarity constraint is introduced to build an online hashing model.

#### 3.3.1. Similarity

Suppose that the newly arrived data at t stage are denoted as $X_{c}^{t}=\left[X_{c1}^{t},X_{c2}^{t},\ldots ,X_{cn_{t}}^{t}\right]$, while the existing data arriving before t stage are denoted as $X_{a}^{t}=\left[X_{a}^{1},X_{a}^{2},\ldots ,X_{a}^{t-1}\right]$. The similarity matrix, $S^{t}$, is constructed by the relationships of the data labels between $X_{c}^{t}$ and $X_{a}^{t}$. Each matrix element is defined as follows:

(7) $$S_{ij}^{t}=\{\begin{array}{cc} 1, & l_{i}^{t}=l_{j}^{t}\\ -1, & otherwise. \end{array}$$

Generally speaking, the more similar the data, the smaller the hash distance. We use the inner product of hash codes to estimate the distance between different vectors in the Hamming space. Constraints on hash codes are constructed using the global similarity matrix which is defined above [35], as shown in Equation (8). Therefore, global semantic information at any stage of the input data remains in the Hamming space. Therefore, the loss function that preserves similarity is defined as follows:

(8) $$L_{2}=\underset{B_{a}^{t},B_{c}^{t}}{\min}{\left\|{B_{c}^{t}}^{T}B_{a}^{t}-kS^{t}\right\|}_{F}^{2}$$

$$s.t.B_{c}^{t}\in {\left\{1,-1\right\}}^{r\times n_{t}},B_{a}^{t}\in {\left\{1,-1\right\}}^{r\times m_{t}}$$

where $m_{t}=\sum_{i=1}^{t-1}n_{i}$ represents the total amount of input data arriving before the t stage, $\|\cdot {\|}_{F}$ refers to the F norm, and k is the bit length of hash codes.

#### 3.3.2. Balanced Similarity

The introduction of a similarity matrix improves the hash codes generated by anchor hashing based on LE, and the global semantic information is better reflected. However, images with different labels among the massive data account for the majority. According to the definition of the global similarity matrix, the value of the element is 1 only when the labels are identical. Therefore, the global similarity matrix is sparse. Data imbalance will cause the loss of similar information, derail the optimization process and eventually drag down the retrieval performance. To solve this issue, we employ the balanced similarity matrix ${\tilde{S}}^{t}$ as follows:

(9) $${\tilde{S}}_{ij}^{t}=\{\begin{array}{cc} \mu_{s}S_{ij}^{t}, & S_{ij}^{t}=1\\ \mu_{d}S_{ij}^{t}, & S_{ij}^{t}=-1, \end{array}$$

where $\mu_{s}$ represents the equilibrium factors of similar pairs, and $\mu_{d}$ represents the equilibrium factors of dissimilar pairs. Usually, we take $\mu_{s}<1$ and $\mu_{d}<1$, which means reducing the Hamming distance of similar vectors and increasing the Hamming distance of dissimilar vectors. By adjusting two equilibrium divisors, the effect that comes from data imbalance is eliminated. By replacing the global similarity matrix, $S^{t}$, in Equation (8) with a balanced similarity matrix, ${\tilde{S}}^{t}$, the loss function that preserves the balanced similarity is defined as follows:

(10) $$L_{2}=\underset{B_{a}^{t},B_{c}^{t}}{\min}{\left\|{B_{c}^{t}}^{T}B_{a}^{t}-k{\tilde{S}}^{t}\right\|}_{F}^{2}$$

$$s.t.B_{c}^{t}\in {\left\{1,-1\right\}}^{r\times n_{t}},B_{a}^{t}\in {\left\{1,-1\right\}}^{r\times m_{t}}$$

### 3.4. Overall Formulation

On one hand, we construct the anchor asymmetric graph to replace the Laplacian graph, preserving the local structural features of the data, and thus obtaining the objective function, $L_{1}$. On the other hand, we perform an inner-product operation on the hash codes of existing data and newly arrived data and then constrain the learning process of hash codes with the global-balanced similarity matrix. The loss function, $L_{2}$, that retains the semantic information of global-balanced similarity is obtained. Under a unified framework, the online hashing preserves both local and global dual-semantic information, and the total loss function $L=L_{1}+L_{2}$ is obtained. By adding a quantized loss function, $L_{3}$, the quantization error between the hash function and the target hash codes is minimized.

(11) $$L_{3}=\underset{W^{t}}{\min\;}{\left\|F\left(X^{t}\right)-B^{t}\right\|}_{F}^{2}$$

The F norm of the projection matrix is used as the penalty term to prevent the model from overfitting. The final objective function is obtained as follows:

(12) $$L=\underset{B_{c}^{t},B_{a}^{t},{\tilde{B}}^{t},W^{t}}{\min}\alpha^{t}\overset{m}{\underset{j=1}{\sum }}\overset{n}{\underset{i=1}{\sum }}{\left\|{\tilde{b}}_{j}^{t}-{W^{t}}^{T}x_{i}^{t}\right\|}_{2}^{2}A_{ij}^{t}+{\left\|{B_{c}^{t}}^{T}B_{a}^{t}-k{\hat{S}}^{t}\right\|}_{F}^{2}\;+\beta^{t}{\left\|{W^{t}}^{T}X_{c}^{t}-B_{c}^{t}\right\|}_{F}^{2}+\gamma^{t}{\left\|W^{t}\right\|}_{F}^{2}$$

where $\alpha^{t}$, $\beta^{t}$, and$\;\gamma^{t}$ are parameters that control the weight of each module.

### 3.5. Alternating Optimization

Because of the binary constraints, Equation (12) is a non-convex objective function in terms of $W^{t}$, $B_{a}^{t}$, $B_{c}^{t}$, ${\tilde{B}}^{t}$. We adopt an alternative optimization approach to deal with the overall formula, L; i.e., and when a variable is updated we assume that the remaining variables are fixed as constants.

- *W^t^*-step: fix $B_{a}^{t}$, $B_{c}^{t}$, ${\tilde{B}}^{t}$, then learn hash weights $W^{t}$. The second term in Equation (12) is eliminated, and the objective function becomes:

(13) $$\underset{W^{t}}{\min}\alpha^{t}\overset{m}{\underset{j=1}{\sum }}\overset{n}{\underset{i=1}{\sum }}{\left\|{\tilde{b}}_{j}^{t}-{W^{t}}^{T}x_{i}^{t}\right\|}_{2}^{2}A_{ij}^{t}+\beta^{t}{\left\|{W^{t}}^{T}X_{c}^{t}-B_{c}^{t}\right\|}_{F}^{2}+{\left\|\gamma^{t}W^{t}\right\|}_{F}^{2}\;$$

We transform and simplify Equation (13) by Equation (14), which reveals the relation between F norm and the trace of a matrix. Then, Equation (15) is obtained.

(14) $$∥A{∥}_{F}=\sqrt{tr\left(A^{T}A\right)\;}=\sqrt{tr\left(AA^{T}\right)\;}$$

(15) $$\underset{W^{t}}{\min\;}\left[\left(\alpha^{t}+\beta^{t}\right)X_{c}^{t}{X_{c}^{t}}^{T}+\gamma^{t}I\right]tr\left(W^{t}W^{t^{T}}\right)-2tr\left(W^{t^{T}}X_{c}^{t}\left(\alpha^{t}A^{t}{{\tilde{B}}^{t}}^{T}+\beta^{t}{B_{c}^{t}}^{T}\right)\right)$$

where I represents the identity matrix of d-dimensional. Equation (15) takes the partial derivative with respect to $W^{t}$, then assigns it zero. We have the following formula:

(16) $$\left[\left(\alpha^{t}+\beta^{t}\right)X_{c}^{t}{X_{c}^{t}}^{T}+\gamma^{t}I\right]W^{t}-X_{c}^{t}\left(\alpha^{t}A^{t}{{\tilde{B}}^{t}}^{T}+\beta^{t}{B_{c}^{t}}^{T}\right)=0$$

Thus, we can get the Equation (17) to update $W^{t}$

(17) $$W^{t}={\left[\left(\alpha^{t}+\beta^{t}\right)X_{c}^{t}{X_{c}^{t}}^{T}+\gamma^{t}I\right]}^{-1}X_{c}^{t}\left(\alpha^{t}A^{t}{{\tilde{B}}^{t}}^{T}+\beta^{t}{B_{c}^{t}}^{T}\right)$$

- $B_{a}^{t}$-step: fix $W^{t}$, $B_{c}^{t}$, ${\tilde{B}}^{t}$, the second term in Equation (12) is retained and the formula becomes:

(18) $$\underset{B_{a}^{t}}{\min}{\left\|B_{c}^{t^{T}}B_{a}^{t}-k{\hat{S}}^{t}\right\|}_{F}^{2}\;$$

According to [49], the L1 norm replaces the F norm, and the result is as follows:

(19) $$B_{a}^{t}=\mathrm{sgn}(B_{c}^{t}{\hat{S}}^{t})\;$$

- $B_{c}^{t}$-step: fix $W^{t}$, $B_{a}^{t}$, ${\tilde{B}}^{t}$, the first and the fourth term in Equation (12) are eliminated, and the corresponding sub-problem is:

(20) $$\underset{B_{c}^{t}}{\min\;}{\left\|B_{c}^{t^{T}}B_{a}^{t}-k{\hat{S}}^{t}\right\|}_{F}^{2}+\beta^{t}{\left\|{W^{t}}^{T}X_{c}^{t}-B_{c}^{t}\right\|}_{F}^{2}$$

In Equation (20), we remove irrelevant terms and the optimization problem becomes:

(21) $$\underset{B_{c}^{t}}{\min}{\left\|B_{a}^{t^{T}}B_{c}^{t}\right\|}_{F}^{2}-2tr\left(P^{T}B_{c}^{T}\right)$$

where tr(·) is trace norm, $P=kB_{a}^{t}{{\hat{S}}^{t}}^{T}+\beta^{t}{W^{t}}^{T}X_{c}^{t}$. In the light of supervised discrete hashing (SDH) [50] and BSODH [35], the solution of Equation (21) becomes NP hard. Therefore, we transfer this issue to row-by-row updating, considering that the matrix is made up of row vectors. Thus, Equation (21) becomes:

(22) $$\underset{{\tilde{b}}_{cr}^{t}}{\min}{\left\|{{\tilde{b}}_{ar}^{t}}^{T}{\tilde{b}}_{cr}^{t}+{{\tilde{B}}_{a}^{t}}^{T}{\tilde{B}}_{c}^{t}\right\|}_{F}^{2}-2tr\left({{\tilde{p}}_{r}^{t}}^{T}{\tilde{b}}_{cr}^{t}+{{\tilde{P}}^{t}}^{T}{\tilde{B}}_{c}^{t}\right)$$

where ${\tilde{b}}_{cr}^{t}$, ${\tilde{b}}_{ar}^{t}$ and ${\tilde{p}}_{r}^{t}$ are the rth row of $B_{c}^{t}$, $B_{a}^{t}$ and ${\tilde{P}}^{t}$, respectively; $B_{c}^{t}$, $B_{a}^{t}$ and ${\tilde{P}}^{t}$ stand for the remaining parts of $B_{c}^{t}$, $B_{a}^{t}$ and ${\tilde{P}}^{t}$ except ${\tilde{b}}_{cr}^{t}$, ${\tilde{b}}_{ar}^{t}$ and ${\tilde{p}}_{r}^{t}$ respectively. Expanding Equation (22), we get the following:

(23) $$\underset{{\tilde{b}}_{cr}^{t}}{\min}{\left\|{{\tilde{b}}_{ar}^{t}}^{T}{\tilde{b}}_{cr}^{t}\right\|}_{F}^{2}+{\left\|{{\tilde{B}}_{a}^{t}}^{T}{\tilde{B}}_{c}^{t}\right\|}_{F}^{2}+2tr\left({{\tilde{B}}_{c}^{t}}^{T}{\tilde{B}}_{a}^{t}{{\tilde{b}}_{ar}^{t}}^{T}{\tilde{b}}_{cr}^{t}\right)-2tr\left({{\tilde{p}}_{r}^{t}}^{T}{\tilde{b}}_{cr}^{t}\right)-\;2tr\left({{\tilde{P}}^{t}}^{T}{\tilde{B}}_{c}^{t}\right)$$

After simplification, the Equation (23) becomes the following:

(24) $$\underset{{\tilde{b}}_{cr}^{t}}{\min}tr\left(\left({{\tilde{B}}_{c}^{t}}^{T}{\tilde{B}}_{a}^{t}{{\tilde{b}}_{ar}^{t}}^{T}-{{\tilde{p}}_{r}^{t}}^{T}\right){\tilde{b}}_{cr}^{t}\right)$$

Therefore, we solve the sub-problem by following updating rules below:

(25) $${\tilde{b}}_{cr}^{t}=sgn\left({\tilde{p}}_{r}^{t}-{\tilde{b}}_{ar}^{t}{{\tilde{B}}_{a}^{t}}^{T}{\tilde{B}}_{c}^{t}\right)$$

- ${\tilde{B}}^{t}$-step: fix $W^{t}$, $B_{a}^{t}$, $B_{c}^{t}$, only the first term remains in Equation (12), and it is transformed into the formula, as follows:

(26) $$\underset{{\tilde{B}}^{t}}{\max}\;tr\left({W^{t}}^{T}X_{c}^{t}A^{t}{{\tilde{B}}^{t}}^{T}\right)$$

Finally, we get the following rule to update the hash codes of the anchor data.

(27) $${\tilde{B}}^{t}=sgn\left({W^{t}}^{T}X_{c}^{t}A^{t}\right).$$

The proposed LSOH is presented in Algorithm 1.
**Algorithm 1** the online hashing preserving local features and global-balanced similarity**Input:** training samples, X; labels L; code length, k; the number of sample batches, T; divisors $\alpha^{t}$,$\beta^{t}$$,\;\gamma^{t}$**.****Output:** hash codes B and the mapping matrix W. Initialize W with the normal Gaussian distribution **while** T←1 **do**  Denote the newly arrived data as $X_{c}^{t}$  Set $X_{a}^{t}=\left[X_{a}^{t};X_{c}^{t}\right]$, $B_{a}^{t}=\left[B_{a}^{t};B_{c}^{t}\right]$  Compute m anchor points $\left[u_{1},u_{2},\ldots ,u_{i},\ldots ,u_{m}\right]$ by means of K-means clustering  Obtain anchor graph $A^{t}$ via Equation (5) and compute the global-balanced similarity matrix ${\tilde{S}}^{t}$ by labels  Update $W^{t}$, $B_{a}^{t}$, ${\tilde{B}}^{t}$ via Equation (17), Equation (19) and Equation (27), **respectively**  **while r** becomes *k*
←1 **do**   Update ${\tilde{b}}_{cr}^{t}$ via Equation (25)   **end while**  **end while** **Set**
$W=W^{t}$ and calculate $B^{t}=sgn\left({W^{t}}^{T}X^{t}\right)$**Return**W**,**B

### 3.6. Computational Complexity

The main computation cost of the iterative algorithm is from the construction of anchor graph A and the optimization of variables. In total, it costs O($Tdn_{t}m$ + $dn_{t}m$) to construct an anchor graph, where T is the iteration times to generate anchors by the clustering algorithm. The time complexities of updating $W^{t},$ $B_{a}^{t}\;$, $B_{c}^{t}\;\mathrm{and}\;{\tilde{B}}^{t}$ at the tth round are O($d^{3}+n_{t}d^{2}+mn_{t}r+n_{t}dr$), O(r$n_{t}m_{t}$), O(km$n_{t}$+ kd$n_{t}+krm+krn_{t}$) and O(dr$n_{t}+mrn_{t}$) respectively. Since d, r, m, $n_{t},\mathrm{and}$ k are much smaller than $m_{t}$, we obtain the time complexity of our proposed LSOH as linear to the size of the data. Obviously, it is scalable to large-scale data.

## 4. Experiments

### 4.1. Datasets

The three datasets used in this paper are CIFAR-10, MNIST, and Places205.

**Figure 2:**
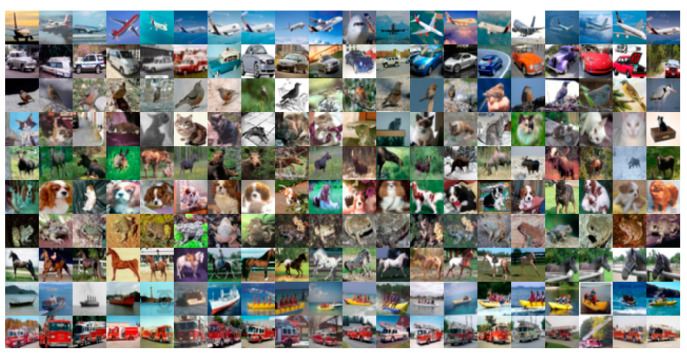
Example images of CIFAR-10 dataset.

CIFAR-10 [51] is a widely recognized dataset. It is made up of 60K samples among 10 classes. Every sample is represented by a 4096-dimensional CNN feature. Following [34], CIFAR-10 is divided into a retrieval set and a test set, where the retrieval set has 59K samples and the test set has 1K samples. The 50K samples within the retrieval set participate in the learning hash function. Twenty example images from each category of CIFAR-10 are shown in Figure 2.

**Figure 3:**
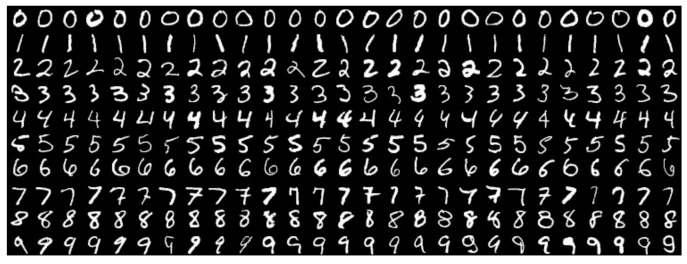
Example images of MNIST dataset.

MNIST is a set of handwritten digit images, with a total of 70 K samples. Every sample is expressed as a 784-dimensional vector. A test set is constructed by sampling 100 samples from each category and the remaining samples form a retrieval set. There are 60 K instances among the retrieval set which participate in the learning hash function. We select 27 example instances randomly from each category, as shown in Figure 3.

**Figure 4:**
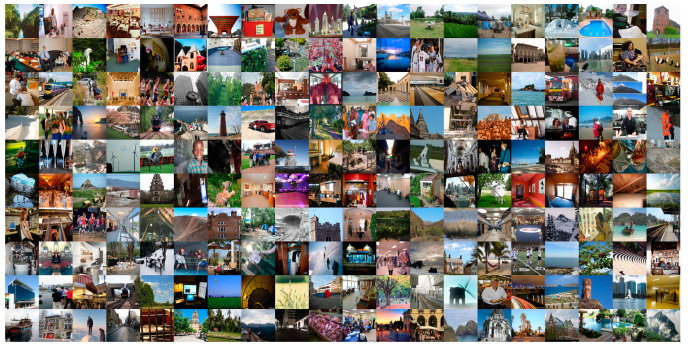
Example images of Places205 dataset.

Places205 has 2.5 million scene images among 205 categories. The instances are firstly processed by the fc7 layer of AlexNet and are fallen to 128-dimensional vectors by PCA. The 20 samples are randomly selected from each category forming the test set. Thus, the rest form the retrieval set. We select a subset of 30K instances randomly from the retrieval set to learn the online hash function. The two hundred images shown in Figure 4 are sampled randomly from Places205.

### 4.2. Experimental Setting

#### 4.2.1. Parameter Setting

Given experience, the scopes of $\alpha^{t}$, $\beta^{t}$, $\gamma^{t}$ for LSOH are set in [0:0.05:1]. For CIFAR-10, the best setting of ($\alpha^{t}$, $\beta^{t}$, $\gamma^{t}$) is empirically adopted to (0.05, 0.6, 0.3). For the MNIST, we set (0.05, 0.6, 0.3) as the configuration of ($\alpha^{t}$, $\beta^{t}$, $\gamma^{t}$). For Places205, (0.05, 0.8, 0.3) corresponds to ($\alpha^{t}$, $\beta^{t}$, $\gamma^{t}$). Table 2 shows the specific parameters of our proposed LSOH on the three datasets mentioned above. We conduct a large number of experiments, where the bit length is taken from the set of [8,16,32,48,64,128]. In addition, the batch size should be greater than that of hash codes in SketchHash [38]. Therefore, the experimental results of SketchHash are presented only when the hashing codes are under 64 bits.

#### 4.2.2. Evaluation Protocols

Some evaluation indicators are adopted, such as the mean average precision (mAP), precision within a Hamming sphere with a radius of 2 centered on every query point (Precision@H2), and the precision of the top-K retrieved neighbors (Precision@K) to evaluate the proposed LSOH. It is worth noting that we apply the average accuracy of the first 1000 retrieved samples (mAP@1000) for Places205, to save calculation time. We adopt the precision–recall (PR) curves on MNIST and CIFAR-10 as well, to compare LSOH and several algorithms.

#### 4.2.3. Compared Methods

To prove the effectiveness of LSOH, we perform abundant experiments and compare LSOH with several advanced OH algorithms such as OKH [24], SketchHash [38], AdaptHash [32], OSH [33], BSODH [35] and DSBOH [52]. The codes of the above comparison methods are publicly available. All the results of the above methods are achieved on a single computer that runs MATLAB and is equipped with a 3.0 GHz Intel Core i5-8500CPU and 16GB RAM. To reduce the error, each experiment was randomly run three times, and then the average is given in this work.

### 4.3. Results and Discussion

The values of mAP and Precision@H2 on the CIFAR-10 dataset are shown in Table 3. It lists the results when generating 8-bit, 16-bit, 32-bit, 48-bit, 64-bit, and 128-bit hash codes under different online methods. The results show that (1) mAP: values of our proposed LSOH are the highest in all the cases. Our proposed LSOH improves the accuracy by 3.3%, 0.4%, 1.9%, 3.4%, 1.5%, and 1.5%, respectively, over the second-best algorithm. It can be seen that LSOH improves the average accuracy, effectively. (2) Precision@H2: our proposed LSOH algorithm is 2.4% higher than the suboptimal algorithm in the situation of 48-bit. It is the second-best algorithm in the case of 8-bit, 16-bit, 32-bit, 64-bit, and 128-bit, while no algorithm can rank first in all the cases. In this way, LSOH still performs well, compared with other algorithms.

Table 4 reveals the values of mAP and Precision@H2 on MNIST. Results show that (1) mAP: In the case of 8-bit, 16-bit, 48-bit, 64-bit, and 128-bit, our proposed LSOH is 2.6%, 0.7%, 0.7%, 0.7%, and 0.8%, respectively, higher than the suboptimal algorithm orderly. It is the second-best algorithm when generating 32-bit hash codes. The advantage of LSOH is verified. (2) Precision@H2: values of Precision@H2 on LSOH are the highest under 8-bit and it ranks second in other code bits. As the bit length grows, the performance of LSOH is worse than that of BSODH under 48-bit, 64-bit, and 128-bit, but better than DSBOH.

The outcomes of mAP and Precision@H2 on the Places205 dataset are expressed in Table 5. (1) mAP: our proposed LSOH is the best algorithm with 3.3% and 1.1% higher than the suboptimal algorithm under 16-bit and 32-bit, respectively. In other cases, the results of LSOH are not optimal. Due to the huge amount of data in Places205, other comparison algorithms have not always performed optimally. In contrast, the LSOH algorithm has better stability and relatively higher retrieval accuracy. (2) Precision@H2: our proposed LSOH has optimal values under 16-bit and 48-bit, with 1.1% and 0.8%, respectively, better than the second-best algorithm, and it ranks second in other code bits. In conclusion, our proposed LSOH algorithm performs well and has high retrieval accuracy on Precision@H2.

**Figure 5:**
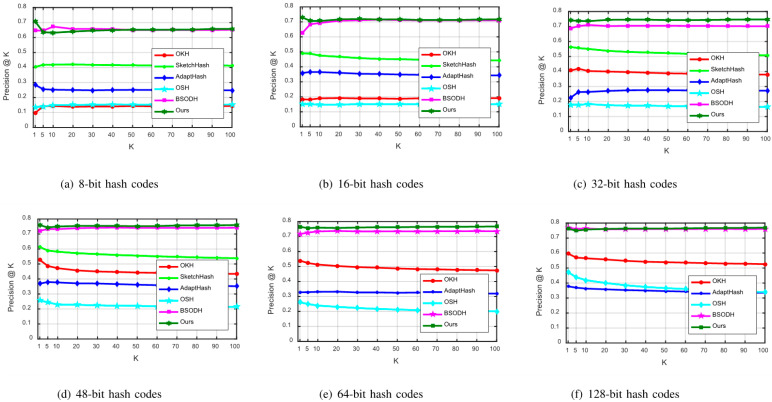
Comparisons of Precision@K curves on CIFAR-10.

**Figure 6:**
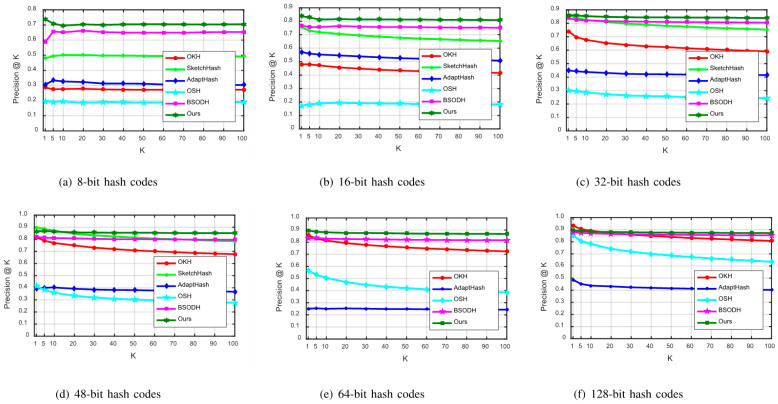
Comparisons of Precision@K curves on MNIST.

For further testing of our proposed LSOH, Precision@K curves in the case of 8-bit, 16-bit, 32-bit, 48-bit, 64-bit, and 128-bit are drawn on CIFAR-10 and MNIST, as displayed in Figure 5 and Figure 6. Comparative experiments of these metrics on the Places205 dataset are not conducted, due to its large memory requirements.

From Figure 5, we can see the Precision@K curves on the CIFAR-10 dataset. It is obvious that the Precision@K curve of our proposed LSOH is higher than other comparison curves in the case of 8-bit, 16-bit, 32-bit, 48-bit, 64-bit, and 128-bit. Thus, the performance of 32-bit and 64-bit hash codes is particularly outstanding. As shown in Figure 6, LSOH continuously reveals a higher Precision@K curve, compared with other algorithms on the MNIST dataset. Only when generating 8-bit hash codes, does our proposed LSOH have a temporary fluctuation on the CIFAR-10 dataset. As hash bits increase, the retrieval accuracy goes up slightly, which shows the robustness and superiority of the LSOH algorithm.

**Figure 7:**
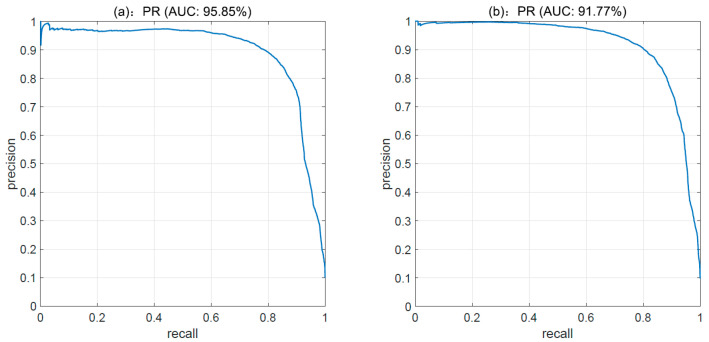
PR curve under 32-bit hash codes. (**a**) PR curve on CIFAR-10 (**b**) PR curve on MNIST.

Figure 7 shows the precision–recall (PR) curves under 32-bit. By calculating the area under curve (AUC) of the PR curves, we obtain the values of 95.85% and 91.77% in turn, which demonstrates that the proposed LSOH has a doubly high ratio of precision and recall.

### 4.4. Parameter Sensitivity

**Figure 8:**
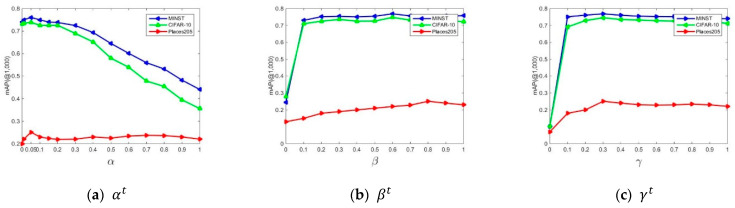
Comparisons of mAP(@1000) performances concerning varying values of $\alpha^{t}$, $\beta^{t}$, $\gamma^{t}$ when the hashing bit is 32.

In this subsection, we conduct the ablation studies on the hyper-parameters of $\alpha^{t}$, $\beta^{t}$, and $\gamma^{t}$, as defined in Equation (12). Without loss of generality, we conduct experiments with varying values of these hyper-parameters concerning mAP(mAP@1000) in the case of 32-bit, in Figure 8. (Detailed values used in this paper are outlined in Table 2.) Similar experimental results can be observed in other hashing bits.

As shown in Equation (12), $\alpha^{t}$ is used to reflect the importance of anchor graph hashing. Figure 8a plots the influence of different values of $\alpha^{t}$ on the performance. Generally speaking, when $\alpha^{t}$ = 0.05 on CIFAR-10 and MNIST, LSOH obtains the best mAP (0.739 on CIFAR-10 and 0.760 on MNIST). When $\alpha^{t}$ = 0.05 on Places205, LSOH obtains the best mAP@1000, with 0.251. Moreover, when $\alpha^{t}$ = 0, LSOH suffers a performance degradation, as can be seen in Figure 8a. More specifically, in this case, the mAP(mAP@1000) scores are 0.740, 0.731, and 0.200 on MNIST, CIFAR-10, and Places205, respectively. To analyze, when $\alpha^{t}$ = 0, LSOH is similar to BSODH. In the experiments, we empirically set the values of $\alpha^{t}$ as 0.05 on all three datasets.

As shown in Equation (12), $\beta^{t}$ is used to reflect the importance of the quantized loss function. From Figure 8b, we can observe that when $\beta^{t}$ = 0.6 on CIFAR-10 and MNIST, LSOH obtains the best mAP (0.768 on MNIST and 0.747 on CIFAR-10). When $\beta^{t}$ = 0.8 on Places205, LSOH obtains the best mAP@1000, with 0.251. Moreover, when $\beta^{t}$ = 0, LSOH suffers great performance degradation, as can be seen in Figure 8b (0.278 on CIFAR-10, 0.244 on MNIST, and 0.13 on Places205). We can observe from Figure 8b that properly applying the quantized loss term in Equation (11) can significantly boost the performance of the three datasets. In the experiments, we empirically set the values of $\beta^{t}$ as 0.6 on CIFAR-10 and MNIST, and 0.8 on Places205.

From Figure 8c, we can observe that when $\gamma^{t}\;$= 0.3 on CIFAR-10 and MNIST, LSOH obtains the best mAP (0.768 on MNIST and 0.745 on CIFAR-10). When $\gamma^{t}\;$= 0.3 on Places205, LSOH obtains the best mAP@1000, with 0.251. Moreover, when $\gamma^{t}\;$= 0, LSOH suffers great performance degradation, as can be seen in Figure 8c. Thus, it is necessary to use a penalty term properly to prevent the model from overfitting. In the experiments, we empirically set the values of $\gamma^{t}$ as 0.3 on the three datasets.

### 4.5. Limitations and Potential Improvements

By comparing the weights of each module in Equation (12), it can be seen that the global-balanced similarity plays an important role in training hash codes. However, some operations on a matrix need to be processed, due to the introduction of anchor hashing, which leads to the training time of LSOH being slightly slower than that of the BSODH algorithm. For example, LSOH takes several seconds longer than BSODH when generating a 32-bit hash code, but it is shorter than OSH. In addition, the inverse of the matrix is required when calculating $W^{t}$, and its time complexity is O($d^{3})$. In other words, it is time-consuming when the dimension of the retrieval image is too large. Therefore, employing a more effective and efficient method to perform the matrix operation is desirable and worthwhile.

## 5. Conclusions

In this paper, a novel hashing algorithm preserving both the local and global dual semantics for image retrieval, i.e. LSOH, was proposed. By extracting the local manifold structure for data coming at the same time, and constructing a global-balanced similarity matrix from data at a different time, we obtain a relatively comprehensive hash constraint, which avoids the problem of over-reliance on labels and imbalanced data updates. Then, an alternative-iteration algorithm is used to solve the discrete binary optimization. Extensive experiments on the benchmark datasets verify that LSOH has significant advantages, compared with other advanced algorithms. However, similar to other state-of-the-art online-hashing algorithms, LSOH decreases the retrieval accuracy with the hash-bits increase. Recently, cross-modal retrieval has had more application requirements, and our method of mining the local structural features of the retrieval data and finding similarity measures of the global data is also worthy of reference and of application in cross-modal retrieval. Given the strong capability for feature representation, the research on online hashing with deep learning networks is also a valuable topic for the future.

## Figures and Tables

**Table 1:** Notations in this paper.

Symbol	Notations
$X^{t}$	input data at t stage
$X_{a}^{t}$	data existing before t stage
$L^{t}$	labels of $X^{t}$
$L_{a}^{t}$	labels of $X_{a}^{t}$
$B^{t}$	hash codes learned for $X^{t}$
$B_{a}^{t}$	hash codes learned for $X_{a}^{t}$.
$W^{t}$	hashing projection matrix at the t age
d	dimension of all input data
$X_{c}^{t}$	newly arrived data at the t stage
***k***	dimension of every hash code
$L_{c}^{t}$	labels of $X_{c}^{t}$
***N***	amount of input data
$B_{c}^{t}$	binary codes generated for $X_{c}^{t}$
$n_{t}$	amount of input data at the t stage

**Table 2:** Parameter settings on three datasets of LSOH.

Parameter	CIFAR-10	MNIST	Places205
$\alpha^{t}$	0.05	0.05	0.05
$\beta^{t}$	0.6	0.6	0.8
$\gamma^{t}$	0.3	0.3	0.3
$\mu_{s}$	1.2	1.2	1
$\mu_{d}$	0.2	0.2	0
$n_{t}$	5000	10,000	10,000

**Table 3:** mAP and Precision@H2 comparisons on CIFAR-10.

	mAP						Precision@H2					
Methods	8-bit	16-bit	32-bit	48-bit	64-bit	128-bit	8-bit	16-bit	32-bit	48-bit	64-bit	128-bit
OKH	0.100	0.134	0.223	0.252	0.268	0.350	0.100	0.175	0.100	0.452	0.175	0.372
SketchHash	0.248	0.301	0.302	0.327	-	-	0.256	0.431	0.385	0.059	-	-
AdaptHash	0.116	0.138	0.216	0.297	0.305	0.293	0.114	0.254	0.185	0.093	0.166	0.164
OSH	0.123	0.126	0.129	0.131	0.127	0.125	0.120	0.123	0.137	0.117	0.083	0.038
BSODH	0.564	0.604	0.689	0.656	0.709	0.711	0.305	0.582	0.691	0.697	**0.690**	**0.602**
DSBOH	0.556	0.669	0.703	0.696	0.720	0.727	**0.411**	**0.730**	**0.737**	0.655	0.552	0.371
Ours	**0.589**	**0.673**	**0.722**	**0.730**	**0.735**	**0.742**	0.366	0.662	0.733	**0.721**	0.675	0.541

The best results are displayed in bold.

**Table 4:** mAP and Precision@H2 comparisons on MNIST.

	mAP						Precision@H2					
Methods	8-bit	16-bit	32-bit	48-bit	64-bit	128-bit	8-bit	16-bit	32-bit	48-bit	64-bit	128-bit
OKH	0.100	0.155	0.224	0.273	0.301	0.404	0.100	0.220	0.457	0.724	0.522	0.124
SketchHash	0.257	0.312	0.348	0.369	-	-	0.261	0.596	0.691	0.251	-	-
AdaptHash	0.138	0.207	0.319	0.318	0.292	0.208	0.153	0.442	0.535	0.335	0.163	0.168
OSH	0.130	0.144	0.130	0.148	0.146	0.143	0.131	0.146	0.192	0.134	0.109	0.019
BSODH	0.593	0.700	0.747	0.743	0.766	0.760	0.308	0.709	0.826	**0.804**	**0.814**	**0.643**
DSBOH	0.596	0.721	**0.759**	0.751	0.781	0.781	0.403	**0.803**	**0.849**	0.788	0.651	0.415
Ours	**0.622**	**0.728**	0.756	**0.758**	**0.788**	**0.789**	**0.418**	0.757	0.846	0.796	0.761	0.487

The best results are displayed in bold.

**Table 5:** mAP@1000 and Precision@H2 comparisons on Places205.

	mAP						Precision@H2					
Methods	8-bit	16-bit	32-bit	48-bit	64-bit	128-bit	8-bit	16-bit	32-bit	48-bit	64-bit	128-bit
OKH	0.018	0.033	0.122	0.048	0.114	0.258	0.007	0.010	0.026	0.017	**0.217**	0.075
SketchHash	**0.052**	0.120	0.202	0.242	-	-	**0.017**	0.066	0.220	0.176	-	-
AdaptHash	0.028	0.097	0.195	0.223	0.222	0.229	0.009	0.051	0.012	0.185	0.021	0.022
OSH	0.018	0.021	0.022	0.032	0.043	0.164	0.007	0.009	0.012	0.023	0.030	0.059
BSODH	0.035	0.174	0.250	0.273	0.308	0.337	0.009	0.101	0.241	0.246	0.212	**0.101**
DSBOH	0.046	0.154	0.240	**0.286**	**0.313**	**0.347**	0.011	0.089	**0.264**	0.175	0.119	0.037
Ours	0.043	**0.187**	**0.251**	0.282	0.296	0.323	0.013	**0.110**	0.244	**0.254**	0.213	0.098

The best results are displayed in bold.

## Data Availability

The data used to support the findings of this study are available from the corresponding author upon request.
